# Syntheses and Self-assembling Behaviors of Pentagonal Conjugates of Tryptophane Zipper-Forming Peptide

**DOI:** 10.3390/ijms12085187

**Published:** 2011-08-15

**Authors:** Kazunori Matsuura, Kazuya Murasato, Nobuo Kimizuka

**Affiliations:** 1 Department of Chemistry and Biochemistry, Graduate School of Engineering, Kyushu University, Moto-oka 744, Nishi-ku, Fukuoka 819-0395, Japan; E-Mails: ma2ra-k@mail.cstm.kyushu-u.ac.jp (K.M.); n-kimi@mail.cstm.kyushu-u.ac.jp (N.K.); 2 International Research Center for Molecular Systems, Kyushu University, Moto-oka 744, Nishi-ku, Fukuoka 819-0395, Japan

**Keywords:** pentagonal conjugate, tryptophane zipper peptide, self-assembly, nanofiber

## Abstract

Pentagonal conjugates of tryptophane zipper-forming peptide (CKTWTWTE) with a pentaazacyclopentadecane core (Pentagonal-Gly-Trpzip and Pentagonal-Ala-Trpzip) were synthesized and their self-assembling behaviors were investigated in water. Pentagonal-Gly-Trpzip self-assembled into nanofibers with the width of about 5 nm in neutral water (pH 7) via formation of tryptophane zipper, which irreversibly converted to nanoribbons by heating. In contrast, Pentagonal-Ala-Trpzip formed irregular aggregates in water.

## 1. Introduction

Multivalent ligand-receptor interactions play pivotal roles in biological systems [1–4]. To date, many artificial multivalent bioconjugates have been developed as inhibitors, receptors, artificial enzymes, signaling molecules, and drug delivery materials. For example, inhibition of Shiga-like or cholera toxins by pentavalent conjugates of oligosaccharides has been reported [5,6]. The concept of “template-assembled synthetic proteins (TASP)” provided a chemical approach to design artificial proteins [7]. Peptide dendrimers have also been developed as multifunctional biomaterials [8,9].

**Figure 1.**
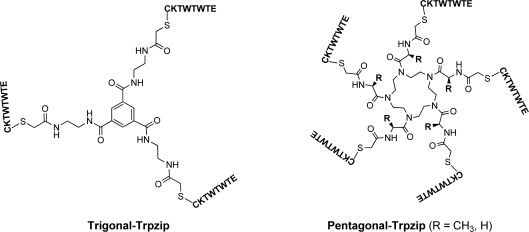
Structures of trigonal and pentagonal conjugates of tryptophane zipper peptide.

Many artificial peptide nano-assemblies have been designed by self-assembly of coiled-coil *α*-helix peptides and *β*-sheet-forming peptides [10–21]. Application of multivalent molecular design to peptide assembly can promote self-assembly and lead to unique morphologies. For example, Ghosh *et al.* reported that PAMAM dendrimer containing four leucine zipper-forming peptides self-assembled into nanofibers [22]. We have designed trigonal peptide conjugates containing *β*-sheet forming peptides [23–25] and glutathione [26–28], and demonstrated that they are useful components for the spontaneous construction of peptide nanoarchitectures in water. Tryptophane zipper has been attracting much attention as a secondary structure motif, which forms stable twisted *β*-hairpin structure due to the interaction between tryptophane residues [29–34]. Recently, we have reported that a novel trigonal peptide conjugate bearing tryptophane zipper-forming peptides showed pH-responding self-assembly into nanospheres and nanofibers (Figure 1) [35].

Most spherical virus capsids are self-assembled from some multiple of 60 chemically identical protein subunits and have an icosahedral symmetry which possesses three- and five-fold rotation axes [36,37]. Molecular design of *C_5_*-symmetric self-assembling molecules would provide chemical strategy for artificial virus capsid, since three-dimensional tiling of pentagon affords dodecahedron. Olson *et al.* demonstrated by molecular dynamics that *C_5_*-symmetric corannulene-based molecules have the potential to self-assemble into dodecahedral nanocapsule [38]. In this paper, we designed a pentagonal peptide conjugate bearing tryptophane zipper-forming peptides (Figure 1), and envisioned that the pentagonal peptide conjugate self-assembles into nanocapsules by formation of intermolecular tryptophane zipper structures. 1,4,7,10,13-Pentaazacyclopentadecane scaffold [6,39] was adopted as a pentagonal core, since the syntheses of pentagonal derivatives are easy to design.

## 2. Results and Discussion

### 2.1. Synthesis of Pentagonal Conjugates of Tryptophane Zipper-Forming Peptide

**Figure 2.**
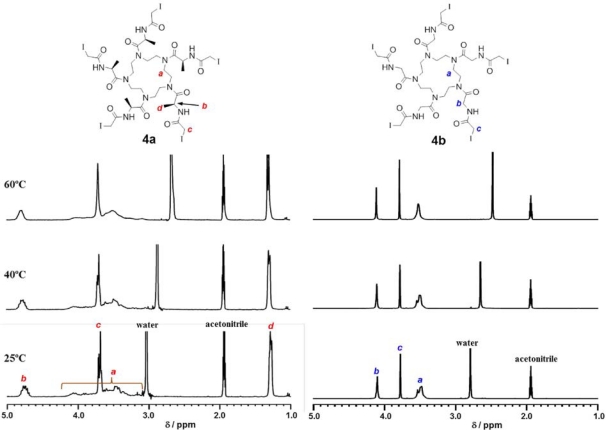
^1^H-NMR spectra of pentagonal iodoacetoamidated core molecules (**4a** and **4b**) at 2.8 mM in mixture of CD_3_CN/D_2_O = 15/1.

**Scheme 1.**
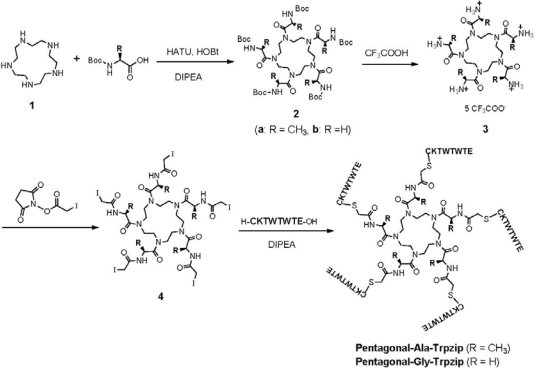
Synthesis of pentagonal conjugates of tryptophane zipper peptide.

1,4,7,10,13-Pentaazacyclopentadecane **1** was synthesized from triethylenetetramine according to the reported procedure [39]. **1** was amidated with Boc-Ala or Boc-Gly, followed by deprotection and iodoacetylation to provide pentagonal core **4** (Scheme 1). ^1^H NMR spectrum of Ala-containing pentagonal core **4a** in mixture of CD_3_CN/D_2_O = 15/1 showed notable broad peaks at the range of *δ* 4.2–3.0 ppm assigned to pentaazacyclopentadecane (*H_a_*) and multiple peaks at *δ* 4.7 (*H_b_*), 3.7 (*H_c_*) and 1.2 ppm (*H_d_*) (Figure 2). The multiple peaks of *H_c_* were unified by elevating temperature, whereas the peaks assigned to *H_a_* were broad even at 60 °C. This indicates that Ala-containing pentagonal core **4a** possesses various irregular conformations of which interconversion are very slow even at 60 °C. In contrast, ^1^H NMR spectrum of Gly-containing pentagonal core **4b** showed relatively sharp peaks (Figure 2), suggesting that **4b** possesses flexible conformation. It is probable that steric hindrance of methyl group of Ala to pentaazacyclopentadecane ring of **4a** prevent flexible interconversion of conformers, whereas steric hindrance in **4b** is smaller than that of **4a**.

The 8-mer peptide h-CKTWTWTE-oh, which was designed based on the intermolecular tryptophane zipper (*β*-hairpin) forming peptide reported by Cohhan *et al.* [29], was synthesized by a standard Fmoc-protected solid-phase method. Pentagonal-Ala-Trpzip and Pentagonal-Gly-Trpzip were prepared by coupling the 8-mer peptides with pentagonal core **4** in the presence of diisopropylethylamine (Scheme 1). These pentagonal conjugates were purified by reverse-phase HPLC and confirmed by MALDI-TOF-MS (*m/z* of Pentagonal-Ala-Trpzip = 6038.96 [M + H]^+^, *m/z* of Pentagonal-Gly-Trpzip = 5970.58 [M + H]^+^). They were soluble in water at a whole range of pH.

### 2.2. Secondary Structure of Pentagonal Peptide Conjugates

**Figure 3.**
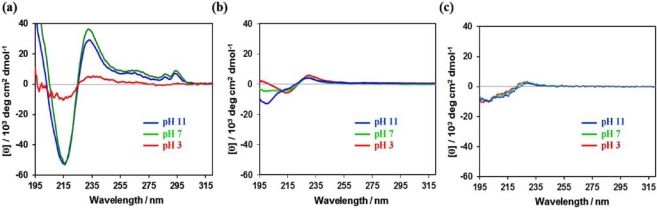
Circular dichroism (CD) spectra of aqueous solution of (**a**) Pentagonal-Gly-Trpzip (10 μM); (**b**) Pentagonal-Ala-Trpzip (10 μM); and (**c**) tryptophane zipper peptide CKTWTWTE (50 μM) at 25 °C in 20 mM citrate buffer (pH 3, red line), in 20 mM phosphate buffer (pH 7, green line), and in aqueous NaOH solution (pH 11, blue line).

After the solutions of pentagonal peptide conjugates were incubated for 48 h at 25 °C in 20 mM citrate buffer (pH 3), in 20 mM phosphate buffer (pH 7), and in aqueous NaOH solution (pH 11), circular dichroism (CD) spectra were measured [40]. It has been reported that tryptophane zipper-forming *β*-hairpin peptide showed exciton-coupling type CD spectrum (positive peak at 230 nm and negative peak at 215 nm) along with small peaks at 285–295 nm based on the interaction between Trp residues [33]. The CD spectra of aqueous solution of Pentagonal-Gly-Trpzip showed negative peak at 216 nm and positive peak at 233 nm together with weak peaks at 280–300 nm in phosphate buffer (pH 7) and aqueous NaOH solution (pH 11) respectively. They indicate the formation of tryptophane zipper and normal *β*-sheet structure, whereas the CD intensity at 216 and 233 nm considerably decreased in citrate buffer (pH 3) (Figure 3(a)). In contrast, the CD spectra of Pentagonal-Ala-Trpzip showed a weak CD pattern at pH 7 and pH 3 which can be ascribed to tryptophane zipper structure, and the content of random coil increased at pH 11 (Figure 3(b)). The difference in secondary structure between these pentagonal Trpzip conjugates might arise from different flexibility of the pentagonal core, as shown by the ^1^H NMR spectra (Figure 2).

The CD spectrum of the precursor peptide CKTWTWTE revealed that the peptide adopted random-coil structure at the pH range of 3–11 (Figure 3(c)). We have previously reported that Trigonal-Trpzip also adopts mixed secondary structures of tryptophane zipper and normal *β*-sheet at pH 7, but the molar elipticity at 216 and 233 nm ([θ]_216_ = −55,000 and [θ]_233_ = 21,000 deg cm^2^ dmol^−1^) was lower than that of Pentagonal-Gly-Trpzip under the same conditions [35]. These results indicate that the formation of tryptophane zipper structure from the peptide CKTWTWTE is promoted by the pentagonal preorganization, which is more effective than the trigonal preorganization.

### 2.3. Self-Assembly of Pentagonal Peptide Conjugates in Water

**Figure 4.**
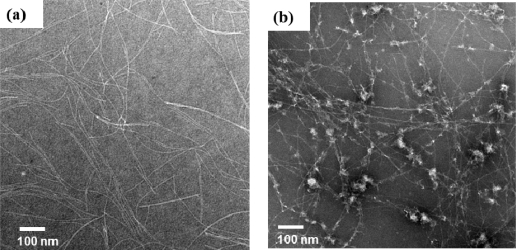
Transmission electron microscopy (TEM) images of pentagonal conjugates of tryptophane zipper peptide (10 μM) in 20 mM phosphate buffer (pH 7) at 25 °C: (**a**) Pentagonal-Gly-Trpzip, and (**b**) Pentagonal-Ala-Trpzip. TEM samples were stained with phosphotungstic acid.

A transmission electron microscopy (TEM) image revealed that Pentagonal-Gly-Trpzip formed only nanofibers of several micrometers length with uniform width of 4–5 nm in phosphate buffer (pH 7, Figure 4(a)), although we envisioned that the conjugate self-assembles into nanocapsules [40]. It is probable that the nanofibers formed by face-to-face assembly of Pentagonal-Gly-Trpzip via parallel tryptophane zipper and *β*-sheet structure. On the other hand, in the TEM image of Pentagonal-Ala-Trpzip, irregular aggregates were observed together with nanofibers (Figure 4(b)). This might reflect less formation of tryptophane zipper structure based on rigid irregular core conformations.

**Figure 5.**
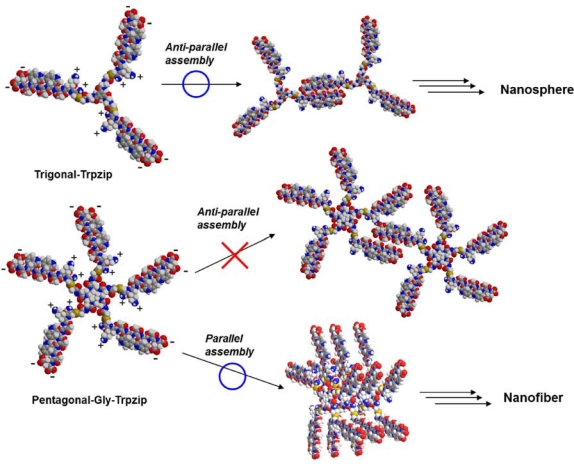
Schematic illustration of the self-assembly of trigonal and pentagonal conjugates of tryptophane zipper peptide.

We have previously reported that Trigonal-Trpzip selectively self-assembled into nanospheres with the size of 20–30 nm at pH 7 [35]. Since the peptide CKTWTWTE might possess zwitterionic structure at pH7, it is reasonable that Trigonal-Trpzip formed nanospheres by forming intermolecular antiparallel *β*-sheet-like structures. In contrast, Pentagonal-Gly-Trpzip selectively formed nanofibers probably due to the formation of parallel *β*-sheet-like structures despite ionic repulsion between peptides (Figure 5). To form nanospheres, it is desirable that the assembly units take concave conformations. The difference in morphology between trignal- and pentagonal-tryptophanezipper conjugtates might arise from difference in the peripheral density of peptide chains and the conformation of scaffold. It seems that steric hindrance among peptide chains in Pentagonal-Gly-Trpzip prevents the formation of anti-parallel *β*-sheet-like structures and concave conformations, in contrast to the case of Trigonal-Trpzip.

**Figure 6.**
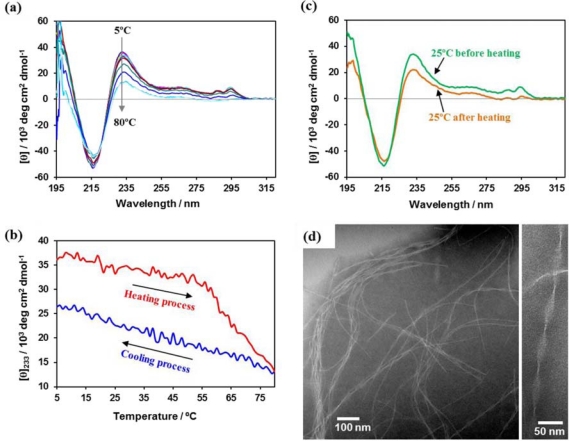
Structural change of Pentagonal-Gly-Trpzip by heating process. (**a**) Temperature dependence of CD spectrum for aqueous solution of Pentagonal-Gly-Trpzip (10 μM) at pH 7; (**b**) Temperature dependence of [θ] at 233 nm for the aqueous solution. The heating and cooling rates were 1 °C/min [41]; (**c**) CD spectra of Pentagonal-Gly-Trpzip at 25 °C before (green line) and after (orange line) the heating process; and (**d**) TEM image of Pentagonal-Gly-Trpzip at 25 °C after the heating process.

Figure 6(a) shows temperature dependence of CD spectrum for aqueous solution of Pentagonal-Gly-Trpzip (10 μM) at pH 7. The positive peaks at 233 and 285–295 nm were gradually decreased by heating process, but the CD intensity at 233 nm hardly recovered by cooling process (Figure 6(b)). CD spectrum of Pentagonal-Gly-Trpzip at 25 °C after the heating process showed formation of normal *β*-sheet-rich structure (Figure 6(c)). These results indicate the irreversible structural change of tryptophane zipper to normal *β*-sheet-rich structure. A TEM image revealed that Pentagonal-Gly-Trpzip formed left-handed helical ribbon structures with the width of 20–30 nm and pitch of 50–100 nm after the heating process (Figure 6(d)). It is probable that kinetically self-assembled nanofibers consisting of tryptphane zipper transformed to thermodynamically stable helical ribbons consisting of normal *β*-sheet by the heating process.

## 3. Experimental Section

### 3.1. General

Reagents were obtained from commercial source and used without further purification. Deionized water of high resistivity (>18 MΩ cm) purified with a Millipore Purification System (Milli-Q water) was used as a solvent of peptide conjugates. ^1^H-NMR spectra were recorded on Bruker AV300M spectrometer. Reversed-phase HPLC was performed at ambient temperature with a Simadzu LC-6AD liquid chromatograph equipped with a UV/Vis detector (220 nm, Shimadzu SPD-10AVvp) using Inertsil ODS-3 (GL Science) or COSMOSIL Protein-R (Nakarai Tesque) columns (250 × 4.6 mm or 250 × 20 mm). MALDI-TOF mass spectra were obtained on Autoflex III (Bruker Daltonics) under the linear/positive mode with *α*-cyano-4-hydroxy cinnamic acid (*α*-CHCA) as matrix.

### 3.2. Synthesis of Peptide Conjugates

1,4,7,10,13-Pentaazacyclopentadecane (**1**): **1** was synthesized from triethylenetetramine according to the reported procedure [39].

Tryptophene zipper-forming peptide (H-CKTWTWTE-OH): Peptide H-Cys(Trt)-Lys(Boc)-Thr(tBu)-Trp(Boc)-Thr(tBu)-Trp(Boc)-Thr(tBu)-Glu(OtBu)-Alko resin was synthesized on α-*p*-alkoxybenzyl alcohol resin (Alko resin, Watanabe Chemical Industries, Ltd., Hiroshima, Japan, 0.69 mmol/g) using standard Fmoc-based FastMoc coupling chemistry (3 eq. Fmoc-amino acids) with an ABI 433A synthesizer (Applied Biosystems, Carlsbad, CA, USA). DMF solution of 2-(*1H*-benzotriazole-1-yl)-1,1,3,3-tetramethyluronium hexafluorophosphate (HBTU, 0.5 M) and 1-hydroxybenzotriazole hydrate (HOBt·H_2_O, 0.5 M) was used as a coupling reagent. 2.0 M diisopropylethylamine (DIPEA) in NMP and 20% piperidine in NMP were used for neutralization and for Fmoc deprotection, respectively. The peptidyl-resin was washed with NMP, dichloromethane and methanol then dried under vacuum. The peptide was deprotected and cleaved from the resin by treatment with a cocktail of TFA/1,2-ethanedithiol/water/triisopropylsilane = 94/2.5/2.5/1 in volume at room temperature for 2 h. The reaction mixture was filtered to remove the resin and the filtrate was concentrated under vacuum. The peptide was precipitated by adding ice-cooled methyl*-tert*-butyl ether to the residue and the supernatent was decanted. After repeating the methyl*-tert*-butyl ether washing 6 times, the precipitated peptide was dried under vacuum. The crude product was purified by reversed-phase HPLC (column: Inertsil ODS-3) eluting with a liner gradient of CH_3_CN/water (18/82 to 30/70 over 60 min) containing 0.1% TFA. The elution fraction containing the desired peptide was lyophilized to give a flocculent solid. The isolated yield was 23%. MALDI-TOF-MS (matrix: α-CHCA): m/z = 1054.47 [M + H]^+. 1^H-NMR (D_2_O, *δ*/ppm): 7.37–7.45 (2 H, m), 7.23–7.28 (2H, m), 6.90–7.08 (6H, m), 4.51 (1H, t, *J* = 7.2 Hz), 4.45 (1H, t, *J* = 7.2 Hz), 4.20 (1H, t, *J* = 7.2 Hz), 4.15 (1H, d, *J* = 5.1 Hz), 4.04–4.10 (3H, m), 3.85–4.01 (4H, m), 3.10 (2H, t, *J* = 6.3 Hz), 2.89–2.97 (4H, m), 2.67 (2H, t, *J* = 7.8 Hz), 2.22 (2H, t, *J* = 7.8 Hz), 1.84–1.99 (1H, m), 1.68–1.84 (1H, m), 1.48–1.58 (2H, m), 1.37–1.48 (2H, m), 1.07–1.20 (2H, m), 0.98 (3H, d, *J* = 6.3 Hz), 0.91 (3H, d, *J* = 6.3 Hz), 0.88 (3H, d, *J* = 6.3 Hz).

*N*,*N″*,*N″*,*N″″*,*N″″*-Pentakis(*N-tert*-butoxycarbonyl-l-alanyl)-1,4,7,10,13-pentaazacyclopentadecane (**2a**): Boc-Ala-OH (265 mg, 1.40 mmol) and *O*-(7-azabenzotriazolyl)-tetramethyluronium hexafluorophosphate (HATU, 558 mg, 1.43 mmol) were dissolved in dry DMF (2.0 mL). DIPEA (244 μL, 1.40 mmol) was added to the mixture by microsyringe, and then the mixture was stirred for 45 min at room temperature. Then a solution of 1,4,7,10,13-pentaazacyclopentadecane **1** (30 mg, 0.14 mmol) in dry DMF (1.0 mL) was added to the mixture. After the mixture was stirred for 43 h at room temperature, the solvent was evaporated under reduced pressure. The residue was dissolved in chloroform and washed with 5% aqueous NaHCO_3_ and deionized water. The organic layer was dried over anhydrous Na_2_SO_4_ and evaporated to a sticky solid. The sticky solid was dissolved in 20% aqueous acetonitrile and was lyophilized to provide a yellowish powder (142 mg). The crude yield was 93%. The product was not purified further and was used in the next reaction. MALDI-TOF-MS (matrix: α-CHCA): m/z = 1092.64 [M + Na]^+. 1^H-NMR (CD_3_OD, *δ*/ppm): 3.4–4.9 (25H, br), 1.48 (45H, br), 1.37 (15H, br).

*N*,*N″*,*N″*,*N″″*,*N″″*-Pentakis(*N-tert*-butoxycarbonylglycyl)-1,4,7,10,13-pentaazacyclopentadecane (**2b**): Compound **2b** was prepared by the almost same procedure described above. The crude yield was 96% (135 mg). The product was not purified further and was used in the next reaction. MALDI-TOF-MS (matrix: α-CHCA): m/z = 1022.94 [M + Na]^+. 1^H-NMR (CD_3_OD, *δ*/ppm): 3.98 (10H, s), 3.5–3.7 (20H, br), 1.340 (45H, br).

*N*,*N″*,*N″*,*N″″*,*N″″*-Penta-l-alanyl-1,4,7,10,13-pentaazacyclopentadecane trifluoroacetic acid salt (**3a**): Trifluoroacetic acid (1.0 mL, 13 mmol) and deionized water were added to a solution of compound **2a** (142 mg, 0.132 mmol) in dichloromethane (2.0 mL). After the mixture was stirred for 1 h at room temperature, excess trifluoroacetic acid and dichloromethane were evaporated under reduced pressure. The residue was washed with methyl*-tert*-butyl ether, and the supernatant was decanted. The residue was dried with argon gas to provide a colorless powder (109 mg). The crude yield was 72%. The product was not purified further and was used in the next reaction. MALDI-TOF-MS (matrix: α-CHCA): m/z = 571.40 [M + H]^+^.

*N*,*N″*,*N″*,*N″″*,*N″″*-Pentaglycyl-1,4,7,10,13-pentaazacyclopentadecane trifluoroacetic acid salt (**3b**): Compound **3b** was prepared by the almost same procedure described above. The crude yield was 100% (123 mg). The product was not purified further and was used in the next reaction. MALDI-TOF-MS (matrix: α-CHCA): m/z = 501.33 [M + H]^+^.

*N*,*N″*,*N″*,*N″″*,*N″″*-Pentakis(*N-*iodoacetyl-l-alanyl)-1,4,7,10,13-pentaazacyclopentadecane (**4a**): A solution of compound **3a** (50 mg, 0.044 mmol) and NaHCO_3_ (37 mg, 0.44 mmol) in deionized water (2.0 mL) was added to a solution of iodoacetic acid *N*-hydroxy succinimide ester (248 mg, 0.876 mmol) in acetone (2.0 mL). The mixture became turbid immediately and was stirred in the dark at room temperature. After 48 h, the reaction mixture indicated acidic pH. The mixture was neutralized with NaHCO_3_ (84 mg, 1.0 mmol) and added iodoacetic acid *N*-hydroxy succinimide ester (50 mg, 0.18 mmol) in acetone (1.0 mL). After the mixture was stirred for 2 h in the dark, the resulted precipitate was filtered. The residue was washed with deionized water and acetone to provide a colorless powder. The crude product was purified by reversed-phase HPLC (column: Inertsil ODS-3) columns eluting with a linear gradient of CH_3_CN/water (30/70 to 50/50 over 20 min). The isolated yield was 12%. MALDI-TOF-MS (matrix: α-CHCA): m/z = 1409.88 [M + H]^+. 1^H-NMR (CD_3_CN + D_2_O, *δ*/ppm): 4.65–4.80 (5H, m), 2.9–4.2 (30H, br m), 1.2–1.3 (15H, m).

*N*,*N″*,*N″*,*N″″*,*N″″*-Pentakis(*N-*iodoacetylglycyl)-1,4,7,10,13-pentaazacyclopentadecane (**4b**): A solution of compound **3b** (47 mg, 0.044 mmol) and NaHCO_3_ (28 mg, 0.33 mmol) in deionized water (1.0 mL) was added to a solution of iodoacetic acid *N*-hydroxy succinimide ester (96 mg, 0.34 mmol) in acetone (1.0 mL). The mixture was stirred for 1 h in the dark at room temperature. After the solvent was evaporated under reduced pressure, the residue was purified by reversed-phase HPLC (column: Inertsil ODS-3) eluting with a linear gradient of CH_3_CN/water (25/75 to 50/50 over 25 min). The isolated yield was 13.4 mg (23%). MALDI-TOF-MS (matrix: α-CHCA): m/z = 1340.79 [M + H]^+. 1^H-NMR (CD_3_CN + D_2_O, *δ*/ppm): 7.5–7.6 (2H, br), 4.10 (10H, s), 3.76 (10H, s), 3.4–3.6 (20H, br m).

**Pentagonal-Ala-Trpzip**: Peptide H-CKTWTWTE-OH (13.8 mg, 10.8 μmol) was dissolved in degassed DMF (4.0 mL) under nitrogen at −20 °C. To the mixture were added a solution of compound **4a** (2.17 mg, 1.54 μmol) in degassed DMF (4 mL) and then a solution of DIPEA (9.4 μL, 54 μmol) in degassed DMF (0.1 mL) at the same temperature in the dark. The mixture was stirred for 4 h under the same conditions. After removal of DMF under reduced pressure, the residue was purified by reversed-phase HPLC (column: COSMOSIL Protein-R) eluting with a linear gradient of CH_3_CN/water (23/77 to 28/72 over 40 min) containing 0.1% TFA. The elution fraction containing the desired conjugate was lyophilized to give a flocculent solid. The isolated yield was 1.8 mg (18%). MALDI-TOF-MS (matrix: α-CHCA): m/z = 6038.96 [M + H]^+^ and 3018 [M + 2H]^2+^.

**Pentagonal-Gly-Trpzip**: Pentagonal-Gly-Trpzip was prepared by the almost same procedure described above and was purified by reversed-phase HPLC (column: COSMOSIL Protein-R) eluting with a linear gradient of CH_3_CN/water (28/72 to 32/68 over 40 min) containing 0.1% TFA. The isolated yield was 1.9 mg (25%). MALDI-TOF-MS (matrix: α-CHCA): m/z = 5970.58 [M + H]^+^.

### 3.3. CD Spectrum Measurements

The stock solutions of pentagonal-peptide conjugates (70 μM, the concentration was determined by absorbance at 280 nm) were prepared by dissolving in aqueous NaOH (pH 11) solution, and then diluted in water, 20 mM phosphate buffer (pH 7), and 20 mM citrate buffer (pH 3), respectively. After the solutions of pentagonal peptide conjugates were incubated for 48 h at 25 °C, CD spectra were taken in a 1.0 mm quartz cell with a JASCO J-820 spectrophotometer equipped with a Peltier-type thermostatic cell holder. Temperature dependence of CD spectrum was recorded at 5, 15, 25, 30, 40, 50, 60, 70, and 80 °C. The heating and cooling rates were 1 °C/min. Before the measurement of the CD spectrum, the solution was pre-incubated for 1 min at each temperature.

### 3.4. Transmission Electron Microscopy (TEM)

A carbon-coated Cu-grid (Oken Co., Ltd., Tokyo, Japan) hydrophilized for 30 sec by hydrophilizing treatment apparatus (JEOL HDT-400). The sample solutions for CD spectra were also used for TEM observation. An aliquot (10 μL) of the solutions was applied to a hydrophilized carbon-coated Cu-grid, left for 60 s, and then removed. Subsequentry, a drop of 2 wt% aqueous phosphotungsic acid was placed on the grids and dried in vacuo (post-staining method). The Cu meshes were subjected to TEM observation (JEOL JEM-2010) with acceleration voltage of 120 kV at 25 °C. All of the measurements were repeated for at least two samples to ensure data reproducibility.

## 4. Conclusions

We have developed pentagonal conjugates of tryptophane zipper-forming peptide with a pentaazacyclopentadecane core (Pentagonal-Gly-Trpzip and Pentagonal-Ala-Trpzip). CD spectra revealed that Pentagonal-Gly-Trpzip formed tryptophane zipper structure at pH7, but Pentagonal-Ala-Trpzip and the precursor peptide CKTWTWTE showed the formation of weak tryptophane zipper or random coil structure. Pentagonal-Gly-Trpzip self-assembled into nanofibers with uniform width at pH 7, whereas Pentagonal-Ala-Trpzip fromed irregular aggregates which reflect less formation of tryptophane zipper structure. The nanofibers from Pentagonal-Gly-Trpzip showed irreversible transformation to helical ribbon accompanying the secondary structural change by heating process. The present peptide nanostructures would be applied as biodegradable nanomaterials and platforms for nano-biotechnology. The present pentagonal molecular design would extend the feasibility of multivalent peptide conjugates.

## References

[1] Mammen M, Choi S, Whitesides GM (1998). Polyvalent interactions in biological systems: Implications for design and use of multivalent ligands and inhibitors. Angew. Chem. Int. Ed.

[2] Kiessling LL, Gestwicki JE, Strong LE (2006). Synthetic multivalent ligands as probes of signal transduction. Angew. Chem. Int. Ed.

[3] Dam TK, Brewer CF (2008). Effects of clustered epitopes in multivalent ligand-receptor interactions. Biochemistry.

[4] Mulder A, Huskens J, Reinhoudt DN (2004). Multivalency in supramolecular chemistry and nanofabrication. Organ. Biomol. Chem.

[5] Kitov PI, Sadowska JM, Mulvey G, Armstrong GD, Ling H, Pannu NS, Read RJ, Bundle DR (2000). Shiga-like toxins are neutralized by tailored multivalent carbohydrate ligands. Nature.

[6] Zhang Z, Merritt EA, Ahn M, Roach C, Hou Z, Verlinde CLMJ, Hol WGJ, Fan E (2002). Solution and crystallographic studies of branched multivalent ligands that inhibit the receptor-binding of cholera toxin. J. Am. Chem. Soc.

[7] Mutter M, Vuilleumier S (1989). A Chemical approach to protein design. Template-assembled synthetic proteins (TASP). Angew. Chem. Int. Ed.

[8] Crespo L, Sanclimens G, Pons M, Giralt E, Royo M, Albericio F (2005). Peptide and amide bond-containing dendrimers. Chem. Rev.

[9] Darbre T, Reymond J-L (2006). Peptide dendrimers as artificial enzymes, receptors, and drug-delivery agents. Acc. Chem. Res.

[10] Zhang S (2003). Fabrication of novel biomaterials through molecular self-assembly. Nat. Biotech.

[11] Gao X, Matsui H (2005). Peptide-based nanotubes and their application in bionanotechnology. Adv. Mater.

[12] Gazit E (2007). Self-assembled peptide nanostructures: The design of molecular building blocks and their technological utilization. Chem. Soc. Rev.

[13] Ryadnov MG, Woolfson DN (2003). Introducing branches into a self-assembling peptide fiber. Angew. Chem. Int. Ed.

[14] Ryadnov MG, Woolfson DN (2003). Engineering the morphology of a self-assembling protein fibre. Nat. Mater.

[15] Smith AM, Acquah SFA, Bone N, Kroto HW, Ryadnov MG, Stevens MSP, Walton DRM, Woolfson DN (2005). Polar assembly in a designed protein fiber. Angew. Chem. Int. Ed.

[16] Boato F, Thomas RM, Ghasparian A, Freund-Renard A, Moehle K, Robinson JA (2007). Synthetic virus-like particles from self-assembling coiled-coil lipopeptides and their use in antigen display to the immune system. Angew. Chem. Int. Ed.

[17] Marini DM, Hwang W, Lauffenburger A, Zhang S, Kamm RD (2002). Left-handed helical ribbon intermediates in the self-assembly of a *β*-sheet peptide. Nano Lett.

[18] Mihara H, Matsumura S, Takahashi T (2005). Construction and control of self-assembly of amyloid and fibrous peptides. Bull. Chem. Soc. Jpn.

[19] Lim Y-B, Lee E, Lee M (2007). Cell-penetrating-peptide-coated nanoribbons for intracellular nanocarriers. Angew. Chem. Int. Ed.

[20] Lim Y-B, Park S, Lee E, Jeong H, Ryu J-H, Lee MS, Lee M (2007). Glycoconjugate nanoribbons from the self-assembly of carbohydrate-peptide block molecules for controllable bacterial cell cluster formation. Biomacromolecules.

[21] Kwon S, Jeon A, Yoo SH, Chung IS, Lee H-S (2010). Unprecedented molecular architectures by the controlled self-assembly of a *β*-peptide foldamer. Angew. Chem. Int. Ed.

[22] Zhou M, Bentley D, Ghosh I (2004). Helical supramolecules and fibers utilizing leucine zipper-displaying dendrimers. J. Am. Chem. Soc.

[23] Matsuura K, Murasato K, Kimizuka N (2005). Artificial peptide-nanospheres self-assembled from three-way junctions of *β*-sheet-forming peptides. J. Am. Chem. Soc.

[24] Murasato K, Matsuura K, Kimizuka N (2008). Self-assembly of nanofiber with uniform width from wheel-type trigonal-*β*-sheet forming peptide. Biomacromolecules.

[25] Matsuura K, Watanabe K, Sakurai K, Matsuzaki T, Kimizuka N (2010). Self-assembled synthetic viral capsids from a 24-mer viral peptide fragment. Angew. Chem. Int. Ed.

[26] Matsuura K, Matsuyama H, Fukuda T, Teramoto T, Watanabe K, Murasato K, Kimizuka N (2009). Spontaneous self-assembly of nano-spheres from trigonal conjugate of glutathione in water. Soft Matter.

[27] Matsuura K, Fujino K, Teramoto T, Murasato K, Kimizuka N (2010). Glutathione nanospheres: Self-assembly of conformation-regulated trigonal-glutathiones in water. Bull Chem Soc Jpn.

[28] Matsuura K, Tochio K, Watanabe K, Kimizuka N (2011). Controlled release of guest molecules from spherical assembly of trigonal-gultathione by a disulfide recombination. Chem. Lett.

[29] Cochran AG, Skelton NJ, Starovasnik MA (2001). Tryptophan zippers: Stable, monomeric *β*-hairpins. Proc. Natl. Acad. Sci. USA.

[30] Richardson JS, Richardson DC (2002). Natural *β*-sheet proteins use negative design to avoid edge-to-edge aggregation. Proc. Natl. Acad. Sci. USA.

[31] Dempsey CE, Piggot TJ, Mason PE (2005). Dissecting contributions to the denaturant sensitivities of proteins. Biochemistry.

[32] Yang WY, Pitera JW, Swope WC, Gruebele M (2004). Heterogeneous folding of the Trpzip hairpin: Full atom simulation and experiment. J. Mol. Biol.

[33] Streicher WW, Makhatadze GI (2006). Calorimetric evidence for a two-state unfolding of the *β*-hairpin peptide Trpzip4. J. Am. Chem. Soc.

[34] Chetal P, Chauhan VS, Sahal D (2005). A meccano set approach of joining trpzip a water soluble *β*-hairpin peptide with a didehydrophenylalanine containing hydrophobic helical peptide. J. Peptide Res.

[35] Matsuura K, Hayashi H, Murasato K, Kimizuka N (2011). Trigonal tryptophane-zipper as a novel building block for pH-responding peptide nano-assemblies. Chem. Commun.

[36] Harrison SC (1984). Multiple modes of subunit association in the structures of simple spherical viruses. Trends Biochem. Sci.

[37] Harrison SC (2001). The familiar and the unexpected in structures of icosahedral viruses. Curr. Opin. Struct. Biol.

[38] Olson AJ, Hu YHE, Kelnan E (2007). Chemical mimicry of viral capsid self-assembly. Proc. Natl. Acad. Sci. USA.

[39] Bencini A, Fabbrizzi L, Poggi A (1981). Formation of nickel(III) complexes with n-dentate amine macrocycles (n = 4, 5, 6). ESR and electrochemical studies. Inorg. Chem.

[40] Extension of the incubation time (96 h) minimally affected to the results of CD spectra and TEM images

[41] The measurement was repeated for three times to confirm data reproducibility. The standard deviation was about 22%.

